# Rituximab Treatment for Nephrotic Syndrome in Children

**DOI:** 10.1007/s40124-014-0065-5

**Published:** 2014-12-06

**Authors:** Kazumoto Iijima, Mayumi Sako, Kandai Nozu

**Affiliations:** 1 Department of Pediatrics, Kobe University Graduate School of Medicine, 7-5-2 Kusunoki-cho, Chuo-ku, Kobe, 650-0017 Japan; 2 Division for Clinical Trials, Department of Development Strategy, Center for Social and Clinical Research, National Research Institute for Child Health and Development, National Center for Child Health and Development, 2-10-1 Okura, Setagaya-ku, Tokyo, 157-8535 Japan

**Keywords:** Rituximab, Nephrotic syndrome, Frequently relapsing, Steroid-dependent, Multicenter, double-blind, randomized, placebo-controlled trial

## Abstract

In the past 10 years, many reports have suggested that rituximab, a chimeric anti-CD20 monoclonal antibody, is effective for children with complicated, frequently relapsing or steroid-dependent nephrotic syndrome (FRNS/SDNS). However, those reports were case reports, case series, retrospective surveys, and single-arm or short-term trials. Therefore, well-designed controlled trials are required to establish the value of rituximab in this condition. To evaluate the efficacy and safety of rituximab in childhood-onset, complicated FRNS/SDNS, a multicenter, double-blind, randomized, placebo-controlled trial was carried out by the Research Group of Childhood-onset Refractory Nephrotic Syndrome (RCRNS) in Japan (RCRNS01). RCRNS01 showed that rituximab is safe and effective for the treatment of childhood-onset, complicated FRNS/SDNS. In 2014, the use of rituximab for patients with complicated FRNS/SDNS was approved, first in the world, by the Ministry of Health, Labour and Welfare, Japan.

## Introduction

Children afflicted with nephrotic syndrome lose proteins to urine, resulting in hypoproteinemia and generalized edema. Idiopathic nephrotic syndrome occurs in two or more children out of 100,000 [1] and is the most common chronic glomerular disease in children. Many patients have minimal change nephrotic syndrome, and most respond well to steroid therapy, but up to half of them develop frequently relapsing nephrotic syndrome or steroid-dependent nephrotic syndrome (FRNS/SDNS) [2]. A total of 10–20 % of idiopathic nephrotic syndrome patients show steroid resistance (steroid-resistant nephrotic syndrome: SRNS), defined as persisting proteinuria after a 4-week course of oral steroids [2]. Standard treatments for FRNS/SDNS are immunosuppressive agents, such as cyclophosphamide, chlorambucil, cyclosporine (CyA), and levamisole, and CyA is often used for treatment of SRNS [3–5]. Most affected children are helped by these drugs; however, some still show complicated clinical courses. A total of 10–20 % of children with FRNS/SDNS on CyA have frequent relapses [6, 7], and approximately 30 % of childhood SRNS patients have steroid-sensitive frequent relapses after achievement of complete remission [8]. In addition, CyA can cause side effects, especially chronic nephrotoxicity [9, 10], suggesting that CyA treatment should be discontinued after its long-term use. However, discontinuing CyA almost always results in frequent relapses or steroid dependence, requiring long-term steroid therapies, which also pose a long-term risk to children. Collectively, at least 10–20 % of children with idiopathic nephrotic syndrome still show frequent relapses or steroid dependence under or after immunosuppressive therapies. We have defined these conditions as “complicated FRNS/SDNS”. Additionally, approximately 2–3 % of children with idiopathic nephrotic syndrome show resistance for steroids and any immunosuppressive agents, which is defined as “refractory SRNS”, posing a high risk of end-stage renal failure (Table 1). Therefore, development of new treatments for complicated FRNS/SDNS and for refractory SRNS is urgently needed.

**Table 1:** Definitions of terms in nephrotic syndrome

Frequent relapsing nephrotic syndrome (FRNS)	Two or more relapses within 6 months after initial remission or 4 or more relapses within any 12-month period
Steroid-dependent nephrotic syndrome (SDNS)	Two consecutive relapses during the reduction of steroid therapy or within 2 weeks of discontinuation of steroid therapy
Steroid-resistant nephrotic syndrome (SRNS)	When the daily administration of prednisolone at 60 mg/m^2^/day does not lead to remission within 4 weeks
Complicated FRNS/SDNS	(1) Diagnosed with frequent relapse (FRNS) or steroid dependence (SDNS) after completion of immunosuppressive drug therapy (such as cyclosporine, cyclophosphamide, mizoribine, or mycophenolate mofetil)
	(2) Diagnosed with frequent relapse (FRNS) or steroid dependence (SDNS) during immunosuppressive drug therapy (such as cyclosporine, cyclophosphamide, mizoribine, or mycophenolate mofetil)
	(3) With a history of steroid resistance and diagnosed with frequent relapse or steroid dependence during or after the completion of immunosuppressive drug therapy (such as cyclosporine or combination of cyclosporine and methylprednisolone)
Refractory SRNS	When the combination of steroids and immunosuppressive agents including calcineurin inhibitors does not lead to remission

## Rituximab Treatment for Nephrotic Syndrome

Rituximab is a chimeric anti-CD20 monoclonal antibody, which inhibits CD20-mediated B-cell proliferation and differentiation, resulting in depletion of peripheral blood B lymphocytes. This drug was developed for the treatment of B-cell non-Hodgkin’s lymphoma and is now indicated for the treatment of patients with autoimmune diseases, including rheumatoid arthritis, Wegener’s granulomatosis, and microscopic polyangiitis [11, 12].

In the past 10 years, there have been anecdotal reports of rituximab being effective for nephrotic syndrome. In 2004, a patient suffering from SDNS complicated with idiopathic thrombocytopenic purpura underwent rituximab treatment, resulting in long-term remission of nephrotic syndrome and idiopathic thrombocytopenic purpura [13]. In 2005, Nozu et al. reported that rituximab treatment induced long-term remission in recurrent nephrotic syndrome and posttransplant lymphoproliferative disorder after renal transplantation [14]. The findings from their report were confirmed by Pescovits et al. in 2006 [15]. However, other reports have shown that none of the patients treated with rituximab achieved remission in recurrent nephrotic syndrome after renal transplantation [16]. Bagga et al. reported three complete and two partial remissions in five patients with refractory SRNS receiving rituximab [17]. Kamei et al. treated 10 children with refractory SRNS with additional rituximab and methylprednisolone pulse therapy. Seven patients achieved complete remission and preserved normal renal function without proteinuria [18]. Although other case reports and case series, as well as the above-mentioned reports, have suggested that rituximab treatment is effective in some patients with refractory SRNS [19–24], there is no evidence that rituximab is effective in patients with refractory SRNS. Indeed, Magnasco et al. reported the results of an open-label, randomized trial including 31 children with refractory SRNS who received calcineurin inhibitors and prednisolone, and 16 of them received an additional two rituximab infusions. However, proteinuria remained unchanged in rituximab-treated patients and none of them had partial or complete remission [25].

Several case reports and case series, as well as survey studies, have suggested that rituximab is effective for patients with complicated (difficult to treat) FRNS/SDNS, allowing discontinuation or reduction steroids and/or immunosuppressants [22, 24, 26–30]. Recent relatively large case series have also shown promising results. Ravani et al. treated 46 children with idiopathic nephrotic syndrome maintained in remission with steroids and calcineurin inhibitors (i.e., complicated FRNS/SDNS) with one to five rituximab courses. They found that the 6-month probability of remission was 48 % after the first remission [31•]. Ruggenenti et al. reported the effects of rituximab therapy followed by immunosuppression withdrawal on disease recurrence in 30 patients (including 10 children) with complicated FRNS/SDNS. In their report, participants received one or two doses of rituximab, and all of them were in remission at 1 year [32•]. Ravani et al. conducted an open-label, randomized, controlled trial to examine the short-term effects of rituximab in children with steroid- and calcineurin-dependent nephrotic syndrome (i.e., complicated FRNS/SDNS). They concluded that rituximab and lower doses of prednisone and calcineurin inhibitors are non-inferior to standard therapy in maintaining short-term remission [33•]. Taken together, these findings suggest that rituximab is effective for children with complicated FRNS/SDNS. However, these studies were case reports, case series, retrospective surveys, and single-arm or short-term trials. Therefore, well-designed controlled trials are required to establish the value of rituximab in this condition.

## A Multicenter, Double-Blind, Randomized, Placebo-Controlled Trial of Rituximab Therapy for Childhood-Onset Complicated FRNS/SDNS

**Fig. 1:**
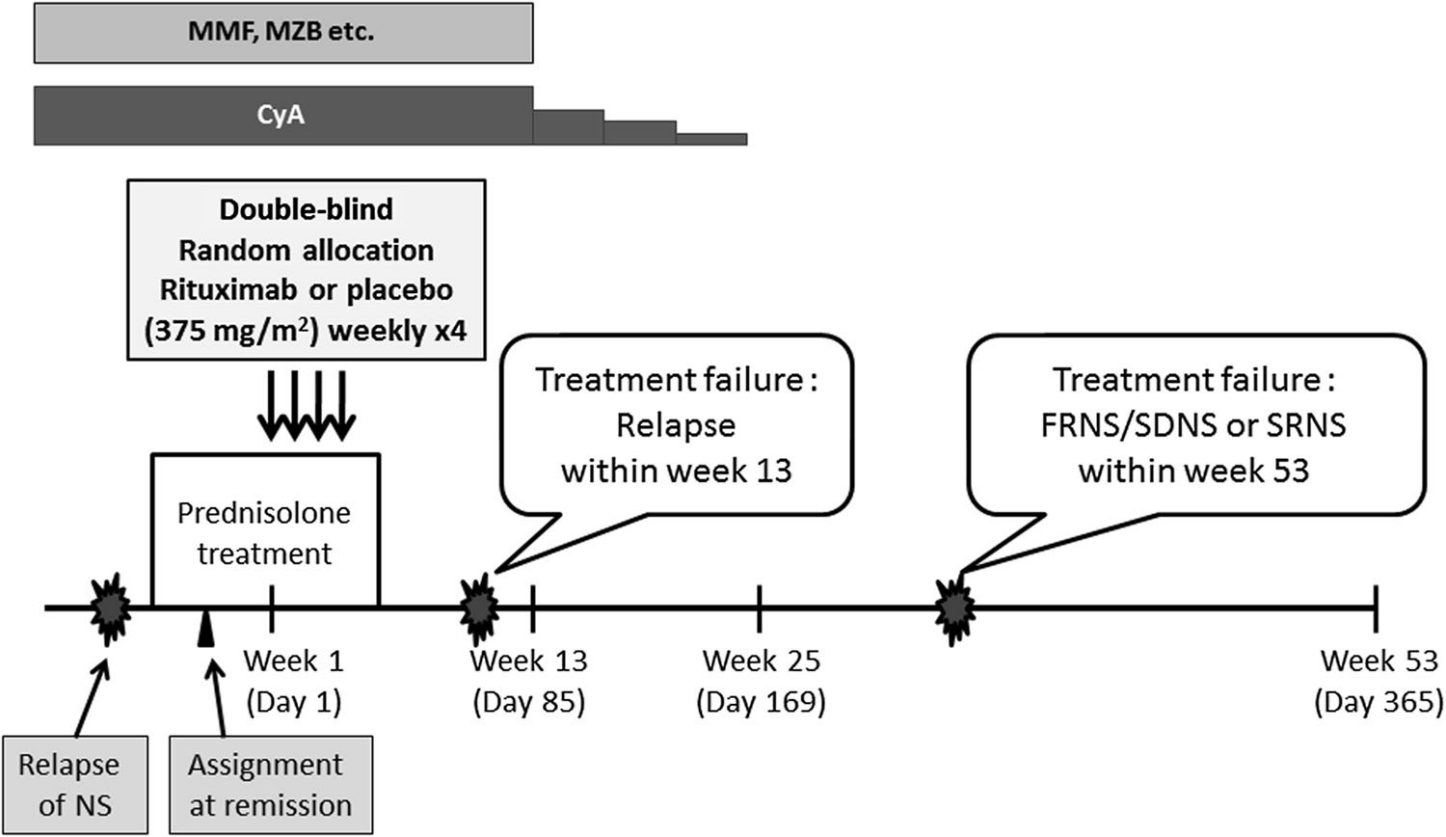
Study design. *NS* nephrotic syndrome, *MMF* mycophenolate mofetil, *MZB* mizoribine, *CyA* cyclosporine

To evaluate the efficacy and safety of rituximab in childhood-onset complicated FRNS/SDNS, a multicenter, double-blind, randomized, placebo-controlled trial was carried out by the Research Group of Childhood-onset Refractory Nephrotic Syndrome (RCRNS) in Japan (RCRNS01) (Clinical Trials Registry ID: UMIN000001405). At the same time, an open-label, multicenter, pharmacokinetic trial (RCRNS-02) (Clinical Trials Registry ID: UMIN000001406) was also carried out. These two trials were investigator-initiated clinical trials, which sought to gain approval from the Ministry of Health, Labour and Welfare, Japan to make rituximab available for patients with childhood-onset complicated FRNS/SDNS. These trials were supported by a Health and Labor Sciences Research Grant for the Large Scale Clinical Trial Network Project (CCT-B-2001). The study design of RCRNS01 is shown in Fig. 1. The protocol for RCRNS01 is available at http://www.med.kobe-u.ac.jp/pediat/pdf/rcrn01.pdf. When patients developed relapse of nephrotic syndrome, they underwent a screening examination and were registered once their eligibility, including steroid sensitivity, was verified. The rituximab group received intravenous rituximab of 375 mg/m^2^ body surface area (maximum 500 mg) once weekly for 4 weeks, and the placebo group received placebo at the same frequency. Prednisolone treatment was gradually discontinued after obtaining remission, and patients were treated with prednisolone when they developed relapses during the study period. Tapering of the CyA dose was started on day 85, and the drug was discontinued by day 169. Other immunosuppressive agents were discontinued by day 85. All of the patients were observed for 1 year, unless the patients dropped out of the study. Patients were considered to have treatment failure if (1) relapse occurred by Day 85, (2) FRNS or SDNS was diagnosed between Day 86 and Day 365, or (3) steroid resistance was diagnosed during the observation period. The primary endpoint was the relapse-free period. The secondary endpoints were time-to-treatment failure, relapse rate, time to FRNS/SDNS, and steroid dose after randomization. Safety endpoints, including frequency and severity of adverse events, were also evaluated. A gold standard, double-blind, placebo-controlled trial was adopted because the use of rituximab in treatment of nephrotic syndrome was not yet approved in any country. In the trial, treatment failures were defined, and in the event that patients had treatment failure, the allocation code was urgently disclosed. If patients were allocated to the placebo group, they were able to enter a separately conducted rituximab pharmacokinetic trial (RCRNS02) after discontinuation or completion of RCRNS01.

Sixty-three patients were screened, and 52 were randomized. Twenty-seven patients were allocated to the rituximab group and 25 to the placebo group. Twenty-four patients in each group (total 48) received the intervention and were included in the analysis on an intention-to-treat basis. Four patients from the rituximab group and 20 from the placebo group had discontinued the intervention, mostly because of treatment failure. However, no patients dropped out of the study before the first relapse (the primary endpoint). All of the patients with treatment failure in the placebo group were enrolled into RCRNS02 after discontinuation (*N* = 18) or completion (*N* = 2) of RCRNS01. Baseline characteristics were similar between the two groups. All of the patients were treated with steroids and/or immunosuppressants at relapse immediately before assignment. Over 70 % of patients reported side effects from steroid treatment.

By the end of the observation period, relapses were reported in 17 patients in the rituximab group and 23 in the placebo group. The 50 % relapse-free period was 267 days [95 % confidence interval (CI) 223–374 days] in the rituximab group and 101 days (95 % CI 70–155 days) in the placebo group. This relapse-free period was significantly longer in the rituximab group than in the placebo group (hazard ratio [HR] = 0.267, 95 % CI 0.135–0.528, *p* < 0.0001). Treatment failure was reported in 10 patients in the rituximab group and 20 in the placebo group. The time-to-treatment failure was significantly longer in the rituximab group than in the placebo group (HR = 0.268, 95 % CI 0.122–0.589, *p* = 0.0005), and the relapse rate was significantly lower in the rituximab group than in the placebo group (1.542 [29/18.81] vs. 4.171 [46/11.03] per person-years, HR = 0.370, 95 % CI 0.231–0.591, *p* < 0.0001). Significantly, fewer patients in the rituximab group experienced frequent relapses or steroid dependence compared with those in the placebo group (HR = 0.169, 95 % CI 0.061–0.464, p = 0.0001). The daily steroid dose after randomization in the rituximab group was significantly lower than that in the placebo group (9.12 ± 5.88 vs. 20.85 ± 9.28 mg/m^2^/day, *p* < 0.0001). The majority of adverse events that were reported were mild and no deaths were reported. Although the rate of serious adverse events was higher in the rituximab group than in the placebo group (42 % [10/24] vs. 25 % [6/24]), but this difference was not significant (Fisher’s exact test, *p* = 0.3587). Mild infusion reactions were reported more frequently in the rituximab group (79 % [19/24]) than in the placebo group (54 % [13/24]), but this difference was not significant (Fisher’s exact test, *p* = 0.1246). No Grade 3 or 4 infusion reactions were reported in either group. In conclusion, rituximab is safe and effective, at least for 1 year, for the treatment of childhood-onset, complicated FRNS/SDNS [34••].

Based on the results from RCRNS01 and RCRNS02, the use of rituximab for patients with complicated FRNS/SDNS was approved, for the first time, by the Ministry of Health, Labour and Welfare, Japan on August 29, 2014.

## Safety of Rituximab

More than 500,000 patients worldwide have received rituximab. Serious adverse events have occurred in only a limited number of these patients, while in the majority of patients, rituximab is safe and well tolerated [35]. However, notably, there have been several reports on serious adverse events related to rituximab. Progressive multifocal leukoencephalopathy is a serious adverse event of rituximab (http://www.fda.gov/safety/medwatch/safetyinformation/safety-relateddruglabelingchanges/ucm123013.htm). Fetal hepatitis by reactivation of hepatitis B virus is also a serious adverse event induced by rituximab [36]. In recent studies of patients with complicated nephrotic syndrome who had been taking rituximab, a pediatric patient died because of pulmonary fibrosis [37]. Kamei et al. also reported that respiratory events, such as cough, bronchospasm, and dyspnea, are relatively common as adverse effects of rituximab [38]. Sellier-Leclerc et al. reported a patient with fulminant myocarditis due to enterovirus who underwent heart transplant surgery [39]. Additional severe adverse effects reported in childhood nephrotic syndrome include Pneumocystis carinii pneumonia [28, 40] and severe immune-mediated ulcerative colitis [41]. These complications might have been underestimated in the literature. Although long-term safety data on anti-CD20 therapy are broadly reassuring, a mortality rate of 3 % has been reported in the 3 years following its initiation in patients with a variety of autoimmune diseases [42], mainly due to infection. The long-term consequences of rituximab infusions in children are not known.

## Mechanisms of Rituximab in Nephrotic Syndrome

The exact pathogenesis of nephrotic syndrome is unknown, but T-cell-mediated immunological abnormalities are thought to play a role [43]. A number of studies have shown that B cells promote T-cell activation, mediate antibody-independent autoimmune damage, and provide co-stimulatory molecules and cytokines, which can sustain T-cell activation in autoimmune diseases [44–47]. Rituximab induces inhibition of B-cell proliferation and B-cell apoptosis [48]. This action leads to B-cell depletion, and thus suppression of B-cell–T-cell interactions, which might prevent recurrence of nephrotic syndrome. Impaired regulatory T (T-reg) cell function in patients with minimal change nephrotic syndrome and induction of remission in nephrotic syndrome by T-reg cells have been previously reported [49–51]. Rituximab may induce an increase in the number and function of T-reg cells [52]. Rituximab-maintained remission in nephrotic syndrome might be due to restoration of T-reg cell function.

Fornoni et al. recently reported that rituximab directly binds to an acid sphingomyelinase-like phosphodiesterase 3b on the cell surface of podocytes, resulting in stability of podocyte structure and function [53]. This may lead to prevention of recurrent focal segmental glomerulosclerosis. Whether a similar mechanism functions in complicated FRNS/SDNS remain to be determined.

## Conclusions and Future Perspectives

Rituximab is a promising option for the treatment of complicated FRNS/SDNS. However, this drug does not cure nephrotic syndrome because all of the patients in the RNRNS01 trial had relapsed by 19 months [34••]. To extend the relapse-free period, further modification of rituximab therapy, including repeated courses and adjunct immunosuppressive therapies, may be necessary. Indeed, a multicenter, double-blind, randomized, placebo-controlled trial to examine the efficacy and safety of mycophenolate mofetil after rituximab therapy for treatment of complicated FRNS/SDNS in children will be started in 2015 in Japan. Moreover, comparison of the efficacy, safety, and cost-effectiveness of various rituximab dosing regimens and B-cell-driven regimens remains to be examined [54]. Further studies are required to examine the long-term effects of rituximab use, particularly in children. A retrospective long-term follow-up study of patients enrolled in RCRNS01 and RCRNS02, focusing on clinical courses, treatments after the clinical trial, growth, and late adverse effects, will be carried out soon in Japan. At present, there is no evidence that rituximab is effective in patients with refractory SRNS. However, Kamei et al. recently reported that additional rituximab combined with conventional methylprednisolone pulse therapy and immunosuppressive agents is a promising option for overcoming refractory SRNS [55]. A multicenter, single-arm trial to examine efficacy and safety of rituximab combined with methylprednisolone pulse therapy and immunosuppressive agents will be started in 2015 in Japan.
